# Is *Colobus guereza gallarum* a valid endemic Ethiopian taxon?

**DOI:** 10.5194/pb-6-7-2019

**Published:** 2019-04-18

**Authors:** Dietmar Zinner, Dereje Tesfaye, Nils C. Stenseth, Afework Bekele, Aemro Mekonnen, Steve Doeschner, Anagaw Atickem, Christian Roos

**Affiliations:** 1 Cognitive Ethology Laboratory, German Primate Center (DPZ), Leibniz Institute for Primate Research, Kellnerweg 4, 37077 Göttingen, Germany; 2 Centre for Ecological and Evolutionary Synthesis (CEES), Department of Biosciences, University of Oslo, P.O. Box 1066 Blindern, 0316 Oslo, Norway; 3 Department of Zoological Sciences, Addis Ababa University, P.O. Box 1176, Addis Ababa, Ethiopia; 4 Gruenstifter, Proskauer Str. 24, 10247 Berlin, Germany; 5 Primate Genetics Laboratory, German Primate Center (DPZ), Leibniz Institute for Primate Research, Kellnerweg 4, 37077 Göttingen, Germany; 6 Gene Bank of Primates, German Primate Center (DPZ), Leibniz Institute for Primate Research, Kellnerweg 4, 37077 Göttingen, Germany

## Abstract

Black-and-white colobus (*Colobus guereza* Rüppell, 1835) are arboreal Old World monkeys inhabiting large parts of the deciduous and evergreen forests of sub-Saharan Africa. Two of the eight subspecies of *Colobus guereza* are endemic to Ethiopia: *C. g. gallarum* and *C. g. guereza*. However, the validity of the Ethiopian taxa is debated and observed morphological differences were attributed to clinal variation within *C. g. guereza*. To date, no molecular phylogeny of the Ethiopian guerezas is available to facilitate their taxonomic classification. We used mitochondrial DNA markers from 94 samples collected across Ethiopia to reconstruct a phylogeny of respective mitochondrial lineages. In our phylogenetic reconstruction, augmented by orthologous sequence information of non-Ethiopian black-and-white colobus from GenBank, we found two major Ethiopian mitochondrial clades, with one being largely congruent with the distribution of *C. g. guereza*. The second clade was found only at two locations in the eastern part of the putative range of *C. g. gallarum*. This second lineage clustered with the lowland form, *C. g. occidentalis*, from central Africa, whereas the *C. g. guereza* lineages clustered with *C. g. caudatus* and *C. g. kikuyuensis* from Kenya and northern Tanzania. These two guereza lineages diverged around 0.7 million years ago. In addition, mitochondrial sequence information does not support unequivocally a distinction of *C. g. caudatus* and *C. g. kikuyuensis*. Our findings indicate a previous biogeographic connection between the ranges of *C. g. occidentalis* and *C. g. gallarum* and a possible secondary invasion of Ethiopia by members of the *C. g. guereza*–*C. g. caudatus*–*C. g. kikuyuensis* clade. Given these phylogenetic relationships, our study supports the two-taxa hypothesis, making *C. g. gallarum* an Ethiopian endemic, and, in combination with the taxon's very restricted range, makes it one of the most endangered subspecies of black-and-white colobus.

## 1 Introduction

The genus *Colobus* comprises five species: *C. satanas*, *C. vellerosus*, *C. polykomos*, *C. angolensis*, and *C. guereza*. The internal taxonomy of the genus is based on morphological features, mainly the extent of the white body markings and the proportion of white fur on the tail and the size of the tail brush (Lydekker, 1905; Schwarz, 1929; Rahm, 1970; Hull, 1978), but also on other traits, such as the acoustic structure of loud calls (“roars”) of adult males (Oates and Trocco, 1983; Oates et al., 2000). A study based on mitochondrial DNA (mtDNA) largely supports the morphology-based phylogeny with one exception: morphologically *C. guereza* appears to be the sister taxon of *C. vellerosus* (Oates and Trocco, 1983), while the molecular study suggests a closer relationship between *C. polykomos* and *C. vellerosus* (Ting, 2008), which makes more sense biogeographically because these two taxa occur parapatrically in western Africa with a possible broad hybrid zone between the Sassandra and Bandama rivers (Groves et al., 1993; Gonedelé Bi et al., 2006). Ting (2008) estimated divergence ages among species between 3.5 and 0.2 million years ago (Ma), with *C. satanas* diverging first followed by *C. angolensis*, *C. guereza*, and most recently *C. vellerosus* and *C. polykomos*.

*C. guereza* is the most widely distributed species of the genus with a more or less continuous range from Gabon and Cameroon in the west to Uganda in the east and a more fragmented distribution in Ethiopia, Kenya, and northern Tanzania (Groves, 2007; Kingdon et al., 2008; Fashing and Oates, 2013). In eastern Africa *C. guereza* is often confined to higher altitudes of isolated mountain ranges, such as Mt Kilimajaro, Mt Kenya, the Aberdare Range, Mt Elgon, or the Matthews Range (Fashing and Oates, 2013).

Several subspecies of *C. guereza* have been described, but they are less strikingly different than those of *C. angolensis* (Groves, 2007). *C. g. caudatus*, the most south-eastern form (Fig. 3e), is very different from the north-western *C. g. occidentalis*, but there is a string of geographically and morphologically intermediate forms (Groves, 2007). Moreover, adaptations to high mountain environments of certain populations contribute additional morphological variation, making subspecies delineation more complicate (Carpateno and Gippoliti, 1994).

Rahm (1970) recognized nine subspecies in *C. guereza*, whereas Dandelot (1971) recognized only six. Hull (1979) conducted a craniometric analysis of 607 skulls (340 males; 267 females) of adult guerezas from 18 populations and recognized eight subspecies (*C. g. guereza*, *C. g. gallarum*, *C. g. occidentalis*, *C. g. dodingae*, *C. g. matschiei*, *C. g. kikuyuensis*, *C. g. percivali*, and *C. g. caudatus*; Table 1). This taxonomy was subsequently adopted by IUCN (Kingdon et al., 2008) and several other authors (Groves, 2001, 2007; Grubb et al., 2003; Anandam et al., 2013; Fashing and Oates, 2013) (see Table 1). Recently, Butynski and de Jong (2018) suggested that *C. g. caudatus* should be elevated to species rank because of its extreme geographic isolation and phenotypical distinctiveness. At least concerning morphology, this suggestion is not in agreement with Hull (1979), who found that *C. g. gallarum* is the most distinctive guereza subspecies. Based on his morphological analysis, Hull (1979) also provided a phylogeny of the subspecies of *C. guereza*, with *C. g. gallarum* as the basal lineage and *C. g. occidentalis* as the most recent. However, to date, no molecular study has been carried out to further clarify the internal phylogeny of *C. guereza*.

**Table 1:** History of the taxonomic classification of *Colobus guereza*.

Rahm (1970)	Dandelot (1971)	Hull (1979)	Groves (2001, 2007)
*Colobus abyssinicus*	*Colobus guereza*	*Colobus guereza*	*Colobus guereza*
*C. a. abyssinicus*	*C. g. guereza* = *C. g. percivali*	*C. g. guereza*	*C. g. guereza*
*C. a. gallarum*	*C. g. gallarum*	*C. g. gallarum*	*C. g. gallarum*
*C. a. occidentalis*	*C. g. occidentalis*	*C. g. occidentalis* = *C. g. uellensis*	*C. g. occidentalis*
*C. a. uellensis*			
*C. a. dodingae*		*C. g. dodingae*	*C. g. dodingae*
*C. a. percivali*		*C. g. percivali*	*C. g. percivali*
*C. a. matschiei*	*C. g. matschiei*	*C. g. matschiei*	*C. g. matschiei*
*C. a. kikuyuensis*	*C. g. kikuyuensis*	*C. g. kikuyuensis*	*C. g. kikuyuensis*
*C. a. caudatus*	*C. g. caudatus*	*C. g. caudatus*	*C. g. caudatus*

Grubb et al. (2003), Fashing and Oates (2013), and Anandam et al. (2013) adopted the taxonomy proposed by Hull (1979) and Groves (2001, 2007).

Two of the subspecies – *C. g. guereza* and *C. g. gallarum* – are possibly endemic to Ethiopia (Fashing and Oates, 2013). *C. g. guereza* is widely distributed from the Omo River valley to the Ethiopian Highlands west, north-west, and south-west of the Rift Valley (Yalden et al., 1977). Its southern distribution limits are not well defined, and they might extend into Kenya (Dandelot, 1971). The distribution of *C. g. gallarum* is even less precisely known. They are reported to be confined to the Ethiopian Highlands, east of the Rift (Gippoliti and Butynski, 2008), but Dandelot (1971) wrote that they occur north-east of the Rift Valley (which is probably wrong) and in the north-eastern part of Oromia, where they occur south-east of the Rift Valley. Fashing and Oates (2013) wrote that *C. g. gallarum* occurs in the Ethiopian Highlands east of the Rift Valley, whereas Carpaneto and Gippoliti (1994) noted striking differences among guerezas east of the Rift Valley, i.e. among guerezas from the Harenna Forest of the Bale Mts and guerezas from the type locality of *C. g. gallarum*.

Earliest records, including a description of the holotype (*Colobus gallarum* Neumann, 1902), were provided by Neumann (1902, p. 3: ”Berge im Quellgebiet des Webbi Shebeli; Gara Mulata bei Harar und Djaffa-Berge im Arussi-Land, ferner Wälder bei Burka auf der Straße von Harar nach Adis Abeba”; ”from mountains near the headwaters of the Webi Shabeelle (Arussi Mts?), from Gara Mulata near Harar, the Djaffa Mountains in Arussi country, and from the forests near Burka along the road from Harar to Addis Abeba”, transl. D. Zinner).

The guerezas of Ethiopia occupy a wide variety of forest habitats such as tropical deciduous forest, montane *Juniperus* and *Hagenia* forest, and riverine forests at altitudes between 400 and 3300 m (Yalden et al., 1977). Since they are not confined to high-altitude habitats, the low-lying Rift Valley most likely does not constitute a dispersal barrier as for other Ethiopian highland species, e.g. Ethiopian wolves *Canis simensis* (Gottelli et al., 2004), geladas *Theropithecus gelada* (Shotake et al., 2016; Zinner et al., 2018a), and several rodent and frog species (Bryjaa et al., 2018; Evans et al., 2011; Freilich et al., 2016).

The classification of *C. g. guereza* and *C. g. gallarum* was mainly based on craniometric differences (Hull, 1979) and on colour differences in their tail bases (Rahm, 1970; Fashing and Oates, 2013). In contrast, Yalden et al. (1977) considered at least the tail colour as a clinal trait, and he noted that no geographic barrier exists between the two populations; thus, he did not accept the subspecific classification. As for the other subspecies of *C. guereza*, no molecular analysis is available for the two Ethiopian taxa.

The aim of our study was a phylogenetic reconstruction of mitochondrial lineages of Ethiopian *C. guereza* to test whether the mtDNA-phylogeny is congruent with the taxonomic distinction between *C. g. guereza* and *C. g. gallarum *as suggested by, e.g., Hull (1979) or whether it supports the clinal hypothesis of Yalden et al. (1977).

## 2 Methods

### 2.1 Ethical statement

Sample collection complied with the laws of Ethiopia and Germany and with the guidelines of the International Primatological Society. During sampling of faecal material, no animals were harmed or disturbed.

### 2.2 Sample collection

**Figure 1:**
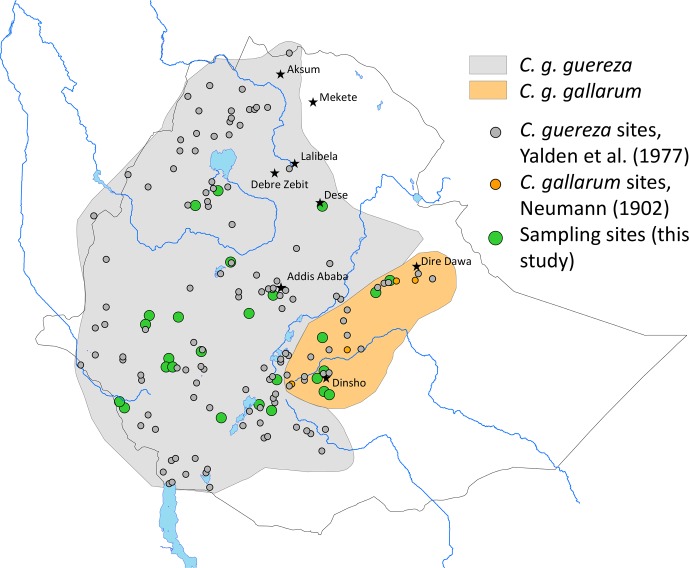
Approximate distribution of *Colobus guereza* subspecies in Ethiopia and sampling sites for genetic analysis (see also Table S1).

We non-invasively collected fresh faecal samples at 26 sites in the range of *C. guereza* (Fig. 1 and Table S1 in the Supplement). Geographic coordinates of each sample were assigned at the time of collection by GPS. Faecal samples were collected and stored following the two-step protocol of Nsubuga et al. (2004) and Roeder et al. (2004). Samples were stored at ambient temperature for up to 3 months in the field and at -20 ${}^{\circ }C$ upon arrival in the laboratory of the German Primate Center (DPZ).

### 2.3 Laboratory methods

We extracted total genomic DNA using the First DNA all tissue kit (Gen-Ial) according to the manufacturer's protocols, with minor modifications as outlined in Kalbitzer et al. (2016). After extraction, DNA concentration was measured with a NanoDrop ND-1000 spectrophotometer (Peqlab) and extracts were stored at -20 ${}^{\circ }C$ until further processing.

We amplified and sequenced two mitochondrial fragments, the complete cytochrome b gene (*cytb*; 1140 bp) and a region spanning a portion of the *NADH* dehydrogenase subunit 3 gene, the tRNA for arginine, the complete *NADH* dehydrogenase subunit 4L gene, and a portion of the *NADH* dehydrogenase subunit 4 gene (*NADH*; 790 bp). We focused on these two markers because for both, several orthologous sequences from *Colobus* are available in GenBank for comparisons.

Cytb was amplified via two overlapping PCR products with sizes of 727 and 663 bp, while *NADH* was amplified via a single PCR product with a size of 873 bp (for primers see Table S2). To minimize the risk of amplifying nuclear mitochondrial-like sequences (numts), primers were specifically designed for *C. guereza* on the basis of available sequence data in Genbank. We conducted PCR reactions in a total volume of 30 µL containing a final concentration of 0.33 µmol of each primer, 3 mmol ${\mathrm{MgCl}}_{2}$, 0166 mmol dNTPs, 1× buffer, 1 U Biotherm Taq DNA polymerase (Genecraft) and 100 ng total genomic DNA. Cycling conditions consisted of pre-denaturation for 2 min at 96 ${}^{\circ }C$, followed by 40 cycles, each with denaturation for 1 min at 96 ${}^{\circ }C$, annealing for 1 min at 50 ${}^{\circ }C$ for both *cytb* fragments and 60 ${}^{\circ }C$ for *NADH*, and extension for 1 min at 72 ${}^{\circ }C$. At the end, a final extension step for 5 min at 72 ${}^{\circ }C$ was added. To check for PCR performance, aliquots of the PCR products were run on 1 % agarose gels. PCR products were cleaned with ExoSAP-IT^™^ Express PCR Product Cleanup Reagent from ThermoFisher and subjected to sequencing using the amplification primers. Sanger sequencing was conducted at GATC Biotech. Sequence electropherograms were checked by eye with 4Peaks 1.8 (https://www.nucleobytes.com, last access: 18 December 2018) and sequences were assembled and manually edited in SeaView 4.4.0 (Gouy et al., 2010). SeaView was also used to check for the correct translation of protein-coding sequences into amino acid sequences.

To reduce cross-sample contamination, all working steps (DNA extraction, PCR set-up, PCR amplification, gel electrophoresis, and PCR product purification) were conducted in separate laboratories. Work benches were cleaned with 10 % bleach and new gloves were used for each sample. Further, PCR controls (without template DNA) were routinely conducted and procedures were repeated for 10 % of randomly selected samples.

### 2.4 Data analysis

We expanded our dataset with orthologous *Colobus* sequences from GenBank (Table S1). We generated three datasets: *NADH* (103 sequences), *cytb* (79 sequences), and the concatenated *NADH* and *cytb* (59 sequences). The number of sequences for each marker differed because (1) for the out-group and most of the non-Ethiopian *C. guereza* taxa, orthologues sequences were not available in GenBank and (2) we were not able to sequence *NADH* and *cytb* from every sample.

For phylogenetic tree reconstructions, identical haplotypes were removed, resulting in datasets with 53 (*NADH*), 34 (*cytb*), and 33 (concatenated) sequences. Phylogenetic trees were constructed with maximum-likelihood (ML) methods using IQ-Tree 1.5.2 (Nguyen et al., 2015) and Bayesian inferences with MrBayes 3.2.6 (Ronquist et al., 2012). The best-fit models for each dataset were determined with ModelFinder (Chernomor et al., 2016; Kalyaanamoorthy et al., 2017) in IQ-Tree under the Bayesian information criterion (BIC). The ML tree was reconstructed with 10 000 ultra-fast bootstrap (BS) replicates (Minh et al., 2013), while the Bayesian tree was obtained from a Markov chain Monte Carlo (MCMC) run with 10 million generations, sampling every 1000 generations. For the Bayesian reconstruction, we checked convergence of all parameters and the adequacy of a 25 % burn-in by assessing the uncorrected potential scale reduction factor (PSRF) (Gelman and Rubin, 1992). Posterior probabilities (PPs) for nodes and a phylogram with mean branch lengths were calculated from the posterior density of trees using MrBayes. Phylogenetic trees were visualized in FigTree 1.4.2 (http://tree.bio.ed.ac.uk/software/figtree/, last access: 18 December 2018).

Divergence times were estimated with a Bayesian approach as implemented in BEAST 2.5.0 (Bouckaert et al., 2014). We performed two independent analyses for each dataset, each with 25 million generations and tree and parameter sampling occurring every 1000 generations. For the analyses, we assumed a relaxed log-normal clock model and applied a coalescent constant population prior for branching rates. As no reliable information from the fossil record is available to calibrate the molecular clock, we used estimated divergence times based on molecular data (Ting, 2008). For both datasets, we set age constraints using a normal prior. For the *NADH* dataset, we constrained the split between *C. satanas* and the other *Colobus* species at a mean of 3.5 with sigma 0.36, translating into a median of 3.5 Ma and a 95 % highest probability density (HPD) of 2.8–4.2 Ma. For the *cytb* and concatenated datasets, we constrained the split between *C. angolensis* and *C. guereza* at a mean of 2.1 with sigma 0.26, translating into a median of 2.1 Ma and a 95 % HPD of 1.6–2.6 Ma. The adequacy of a 10 % burn-in and convergence of all parameters was assessed by inspecting the trace of the parameters across generations using Tracer 1.6 (http://beast.bio.ed.ac.uk/Tracer, last access: 18 December 2018). Sampling distributions of the run were combined with LogCombiner 2.5.0, and the tree with mean node heights was summarized with TreeAnnotator 2.5.0 using a burn-in of 10 %. The trees were visualized in FigTree.

**Figure 2:**
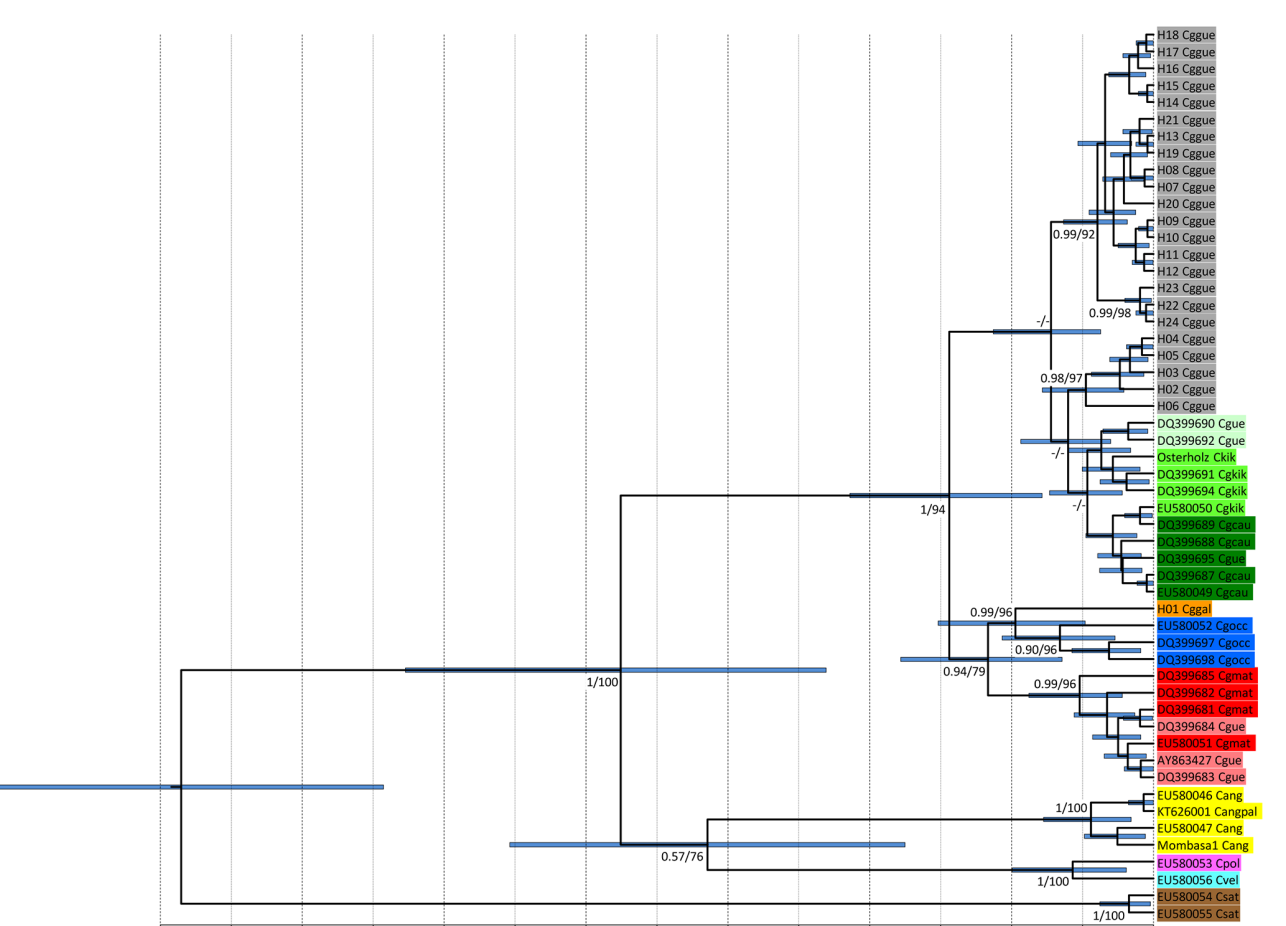
Ultrametric tree showing phylogenetic relationships and divergence times of *Colobus* mtDNA lineages (based on 790 bp of *NADH*). Tip labels refer to *Colobus* haplotypes (see Table S1). Csat: *Colobus satanas*; Cvel: *C. vellerosus*; Cpol: *C. polykomos*; Cang: *C. angolensis*; Cgocc: *C. guereza occidentalis*; Cggal: *C. g. gallarum*; Cgmat: *C. g. matschiei*; Cggue: *C. g. guereza*; Cgkik: *C. g. kikuyuensis*; Cgcau: *C. g. caudatus*; Cgue: *C. guereza* ssp. Node labels refer to ML BS and Bayesian PP support values. The timescale below the tree indicates million years BP.

## 3 Results

For *NADH* we retrieved 74 sequences containing 24 haplotypes, and for *cytb* we retrieved 76 sequences including 31 haplotypes, resulting in 56 concatenated sequences including 30 haplotypes. Independently of the markers used and algorithms applied, the phylogenetic reconstructions show the same tree topology (Figs. 2, S1–S3 in the Supplement). The specimens of the eastern part of the original *C. g. gallarum* range have highly distinct haplotypes, compared to specimens from the range of *C. g. guereza*. The phylogeny based on *NADH* (for which we were able to include sequences of several other *Colobus* taxa) revealed that the supposed *C. g. gallarum* haplotypes cluster with supposed *C. g. occidentalis* (Fig. 2). The combined *C. g. gallarum* + *C. g. occidentalis* clade clusters with a clade containing supposed *C. g. matschiei*. These three taxa form a clade which is the sister clade of a mixed clade of *C. g. caudatus*, *C. g. kikuyuensis*, and *C. g. guereza*. The overall *Colobus* phylogeny revealed the basal position of *C. satanas*, forming the out-group to all other *Colobus* linages (Fig. 2). At around 1.8 Ma a combined clade of the western African taxa (*C. vellerosus* and *C. polykomos*) and *C. angolensis* split off from *C. guereza*. Within *C. guereza* the first divergence appeared at around 0.7 Ma, when the lineage leading to *C. g. occidentalis*, *C. g. gallarum*, and *C. g. matschiei* separated from the lineage leading to *C. g. caudatus*, *C. g. kikuyuensis* and *C. g. guereza*. *C. g. matschiei* split off from the *C. g. occidentalis* + *C. g. gallarum* clade at around 0.6 Ma, which further diverged at around 0.5 Ma. *C. g. caudatus* and *C. g. kikuyuensis* separated only at around 0.2 Ma. The phylogenetic relationships among *C. g. caudatus*, *C. g. kikuyuensis* and *C. g. guereza* are not well resolved, most likely because they diverged relatively recently (<0.3 Ma).

The geographic pattern of the distribution of *C. guereza* haplotypes in Ethiopia revealed that the supposed *C. g. gallarum* haplotype was only found at two sites in the eastern part of the *C. g. gallarum* range whereas those specimens from the western part of the range (e.g. Arsi [Arusi, Arussi] Mts) cluster with *C. g. guereza*.

## 4 Discussion

Our study reveals that mitochondrial haplotypes of *C. guereza* form two major clades which separated around 0.7 Ma. One clade contains haplotypes of *C. g. occidentalis*, *C. g. gallarum*, and *C. g. matschiei*, the other haplotypes of *C. g. caudatus*, *C. g. kikuyuensis*, and *C. g. guereza*. The phylogenetic relationships among the latter three taxa are not resolved, which is most likely a result of their recent divergence where lineage sorting is still incomplete. A complete phylogenetic analysis of all supposed taxa of *C. guereza* was not possible because haplotypes of *C. g. dodingae* and *C. g. percivali* were not available.

Two major mitochondrial lineages occur in Ethiopia, and their geographic distribution largely corresponds to the geographic ranges of *C. g. guereza* and *C. g. gallarum* (Fig. 1). However, the actual range of the *C. g. gallarum* haplotype is much smaller than the range of *C. g. gallarum* as depicted in, e.g., Fashing and Oates (2013). The *C. g. gallarum* haplotype was found only in two local populations in the eastern part of the assumed range of *C. g. gallarum*, whereas in the western part of the range (Arussi Mts), we found only *C. g. guereza* haplotypes. This supports the notion of Carpaneto and Gippoliti (1994) that guerezas of the western part (e.g. Harenna Forest) are phenotypically different from *C. g. gallarum*. The Rift Valley does not constitute a boundary between *C. g. gallarum* and *C. g. guereza* haplotypes, contradicting the notion by Fashing and Oates (2013) that *C. g. guereza* is confined to the west of the Rift Valley.

### 4.1 Taxonomy

In primates, as in other taxonomic groups, taxon delimitation based on mitochondrial sequence information often remains unreliable if the respective divergences or radiations are relatively recent. Often mitochondrial clades mirror biogeographic patterns rather than taxonomy (e.g. baboons; Zinner et al., 2009, 2015, 2018b). Since our study was based on mitochondrial markers, we thus have to be cautious when discussing possible taxonomic inferences.

However, results of our study principally support the two-taxa hypothesis for Ethiopian black-and-white colobus. Notably the two Ethiopian mitochondrial lineages' membership of to two distinct *C. guereza* clades that separated at around 0.7 Ma somehow indicates independent evolutionary histories of the putative subspecies. This divergence age is even more remarkable as the divergence ages among *C. g. guereza*, *C. g. caudatus*, and *C. g. kikuyuensis* are much younger (<0.3 Ma). Also, the phylogenetic relationships among the three taxa, in particular between *C. g. caudatus* and *C. g. kikuyuensis* are not well resolved and only weakly supported (Fig. S1a and S1b). Therefore, mitochondrial sequence information also does not provide evidence for *C. g. caudatus* being the most distinctive form of black-and-white colobus subspecies and thus, do not support the proposal by Butynski and de Jong (2018) to elevate *C. g. caudatus* to a separate species (*C. caudatus*). However, at the moment our taxonomic inferences regarding non-Ethiopian *C. guereza* have to be preliminary since the geographic provenance of several mtDNA sequences is from zoo animals, and we have to rely on the taxonomic classification provided by the authors of the respective sequences.

Since hybridization and introgression is common among various primate taxa (Zinner et al., 2011; Cortes-Ortiz et al., 2019), the Ethiopian guerezas might also be affected, which would partially explain the different views regarding their taxonomy and delineation of subspecies (Yalden et al., 1977; Fashing and Oates, 2013). A thorough study based on genomic data and a reassessment of their morphology would help to shed light on the internal phylogeny of *C. guereza*.

### 4.2 Phylogeography

The close phylogenetic relationship of the eastern Ethioipian *C. g. gallarum* with the central African lowland *C. g. occidentalis* was unexpected. *C. guereza* is ecologically relatively flexible (Fashing, 1999; Fashing and Oates, 2013; Harris and Chapman, 2007) but, as an arboreal species, depends on forest or woodland. Given the historical changes in climate and forest cover in Africa (deMenocal, 2004; Trauth et al., 2009; Hoag and Svenning, 2017), one can assume that ancestral *C. guereza* populations became isolated in forest refugia under unfavourable conditions, whereas forests and *C. guereza* populations became reconnected under favourable conditions.

**Figure 3:**
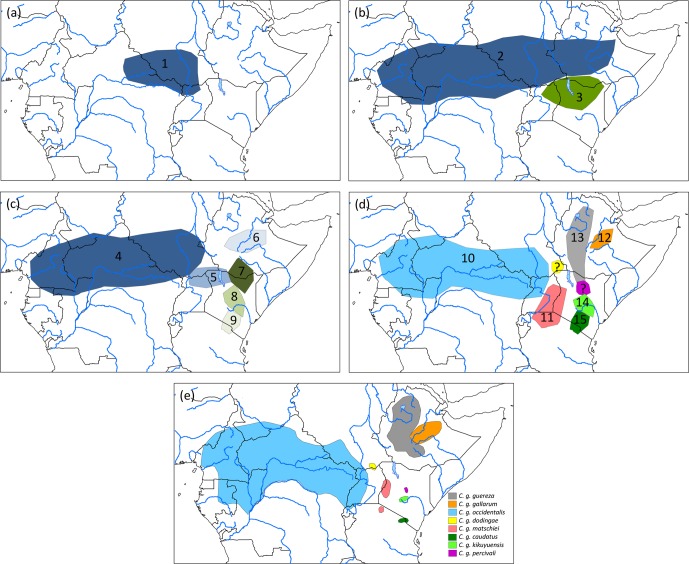
Phylogeographic scenario for *Colobus guereza*. **(a)** Ancestral population (1) in the northern forest belt; **(b)** distribution of two subpopulations after the first split of the ancestral lineage into ancestral populations of *C. g. occidentalis* + *C. g. matschiei* + *C. g. gallarum* (2) and *C. g. caudatus* + *C. g. kikuyuensis* + *C. g. guereza* (3); **(c)** further split of population (2) into ancestral *C. g. occidentalis* (4), *C. g. matschiei* (5), and *C. g. gallarum* (6) populations and split of population (3) into ancestral *C. g. guereza* (7), *C. g. kikuyuensis* (8), and *C. g. caudatus* (9) populations; **(d)** further differentiation of ancestral populations and northern range expansion of *C. g. guereza* into Ethiopia; the phylogenetic relationships of *C. g. percivali* and *C. g. dodingae* have to currently remain open because relevant mtDNA sequences are not available; **(e)** current distribution of *C. guereza* taxa modified after Fashing and Oates (2013).

Assuming an origin of *C. guereza* in the northern forest belt of central Africa, a possible phylogegeographic scenario (Fig. 3) could have started with a range expansion into the forests of north-east Africa and Ethiopia, followed by a first divide into the more central and northern ancestral population of the *C. g. occidentalis* + *C. g. matschiei* + *C. g. gallarum* clade and more southern population of *C. g. guereza* + *C. g. kikuyuensis* + *C. g. caudatus* clade. Both populations further radiated into the respective subspecies. Under unfavourable conditions, *C. g. gallarum* became isolated in the extreme eastern part of the range. The gap between the eastern and more western and central distribution was then probably filled by a range expansion of the southern clade. In order to find support for such a scenario, genetic analyses of the missing populations of *C. g. dodingae* and *C. g. percivali* would be helpful. One could expect clustering of *C. g. dodingae* with *C. g. occidentalis* or *C. g. matschiei*, which would narrow the gap towards *C. g. gallarum* and clustering of *C. g. percivali* with *C. g. kikuyuensis* or *C. g. guereza*, supporting a south–north expansion into the gap.

### 4.3 Conservation

If the distinctiveness of the populations carrying *C. g. gallarum* haplotypes is supported by morphological and nuclear characters, *C. g. gallarum* is most likely the most threatened subspecies of *C. guereza*. At the moment the *C. g. gallarum* haplotypes are only found at two sites where small populations may subsist. Since the forests in the supposed former range of *C. g. gallarum* have been heavily reduced, the outlook for this taxon is not promising (Gippoliti and Butynski, 2008). In contrast, *C. g. guereza* is still widely distributed in Ethiopia. Since both Ethiopian guereza taxa are closely related, there is a certain danger of hybridization if they meet. It would probably be interesting, whether genetic traces of earlier hybridization can be found in *C. g. gallarum*. At the moment, the *C. g. gallarum* populations seem to be geographically isolated from the closest *C. g. guereza* population, but care should be taken to prevent the restocking of the small *C. g. gallarum* population with *C. g. guereza* individuals, even for conservation reasons (Gippoliti et al., 2018).

It also becomes obvious that, if genetic diversity and local adaptation across the complete range are to be preserved (Harcourt, 2006; Harcourt et al., 2005), several subspecies of *C. guereza* need particular conservation measures, including *C. g. gallarum*.

## 5 Conclusion

Our mitochondrial phylogeny revealed that two mitochondrial clades exist in Ethiopian guerezas and that their respective distributions are largely congruent with the geographic ranges of *C. g. gallarum* and *C. g. guereza*. The phylogenetic relationships of the two mitochondrial clades indicate that the two subspecies are not sister taxa. In contrast, our findings indicate a previous biogeographic connection between the ranges of *C. g. occidentalis* and *C. g. gallarum* and a possible secondary invasion of Ethiopia by members of the *C. g. guereza*–*C. g. caudatus*–*C. g. kikuyuensis* clade. Given these phylogenetic relationships, our study supports the two-taxa hypothesis, and in combination with its very restricted range, makes *C. g. gallarum* one of the most endangered subspecies of black-and-white colobus. However, to fully understand the phylogenetic relationship among *C. guereza* populations, a reassessment of their morphology might be helpful, and in any case, a study using nuclear markers is needed.

## Supplement

10.5194/pb-6-7-2019-supplement The supplement related to this article is available online at: https://doi.org/10.5194/pb-6-7-2019-supplement.

## Data Availability

Sequence data are available from NCBI GenBank ().
