# Supplementary material for: Is Colobus guereza gallarum a valid endemic Ethiopian taxon?
Source: Primate Biol. 2019 Apr 18;6(1):7–16. doi: 10.5194/pb-6-7-2019 (PMC7041534; doi:10.5194/pb-6-7-2019)
Supplement: The supplement related to this article is available online at: https://doi.org/10.5194/pb-6-7-2019-supplement. [file pb-6-7-supplement.pdf]

Supplement of Primate Biol., 6, 7–16, 2019
https://doi.org/10.5194/pb-6-7-2019-supplement
© Author(s) 2019. This work is distributed under
the Creative Commons Attribution 4.0 License.

Primate Biology
Open Access

Supplement of

Is *Colobus guereza gallarum* a valid endemic Ethiopian taxon?

Dietmar Zinner et al.

Correspondence to: Dietmar Zinner (dzinner@dpz.eu)

The copyright of individual parts of the supplement might differ from the CC BY 4.0 License.

Supplement Material

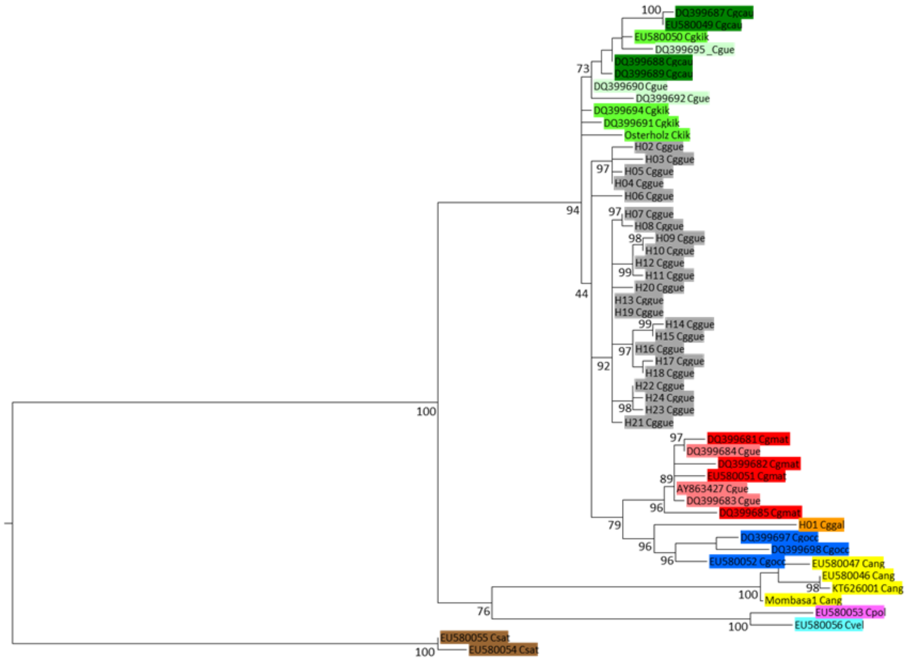

Figure S1a. ML tree (NADH). Csat = *Colobus satanas*, Cvel = *C. vellerosus*, Cpol = *C. polykomos*, Cang = *C. angolensis*, Cgocc = *C. guereza occidentalis*, Cggal = *C. g. gallarum*, Cgmat = *C. g. matschiei*, Cggue = *C. g. guereza*, Cgkik = *C. g. kikuyuensis*, Cgcau = *C. g. caudatus*, Cgue = *C. guereza* ssp.. Node labels refer to ML BS values.

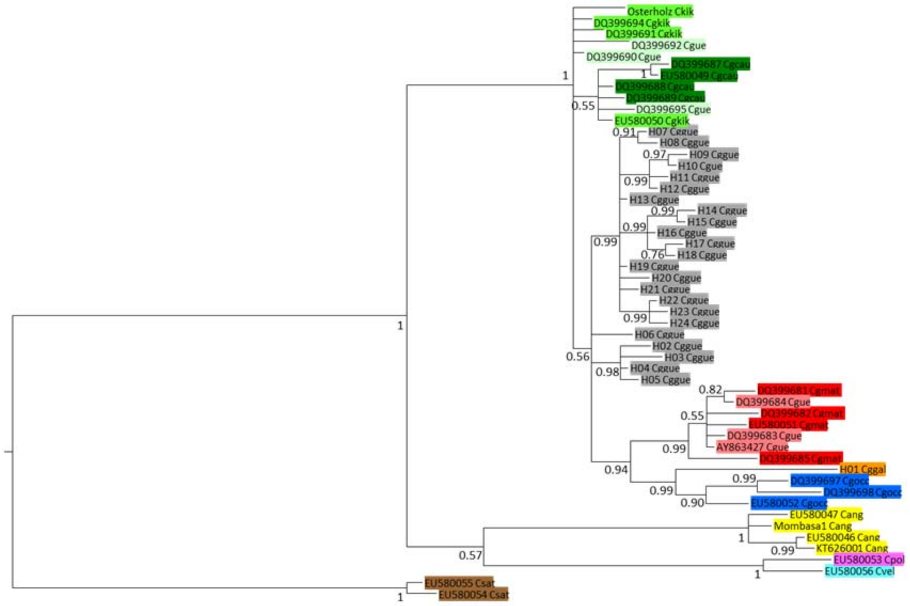

Figure S1b. Bayesian tree (NADH). Csat = *Colobus satanas*, Cvel = *C. vellerosus*, Cpol = *C. polykomos*, Cang = *C. angolensis*, Cgocc = *C. guereza occidentalis*, Cggal = *C. g. gallarum*, Cgmat = *C. g. matschiei*, Cggue = *C. g. guereza*, Cgkik = *C. g. kikuyuensis*, Cgcau = *C. g. caudatus*, Cgue = *C. guereza* ssp.. Node labels refer to Bayesian PPs.

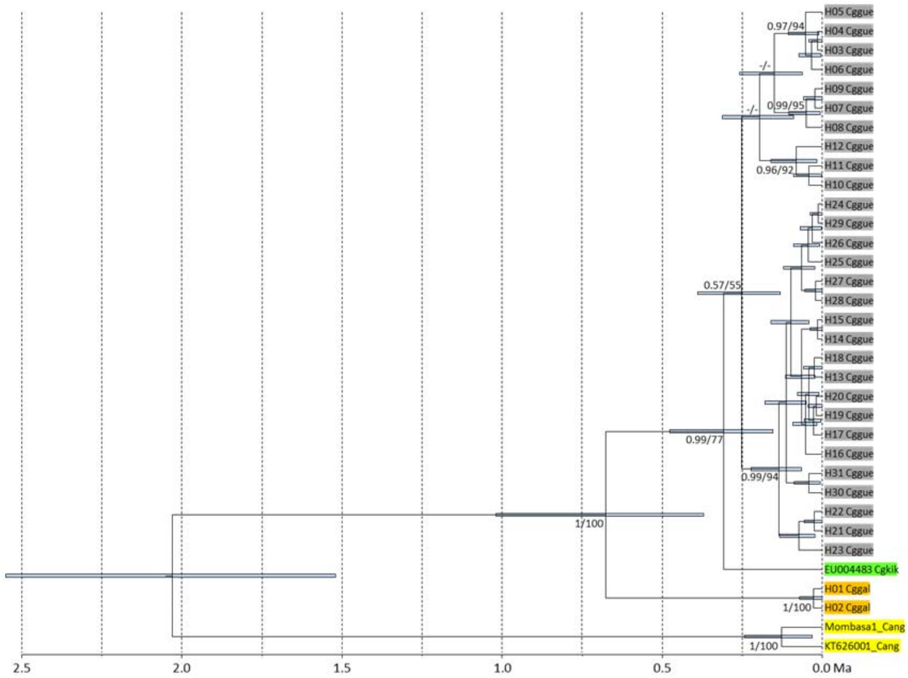

Figure S2a. Ultrametric tree showing phylogenetic relationships and divergence times of *Colobus* mtDNA lineages (based on 1140 bp of cytb). Tip labels refer to *Colobus* haplotypes (see Table S1). Cang = *C. angolensis*, Cggal = *C. g. gallarum*, Cggue = *C. g. guereza*, Cgkik = *C. g. kikuyuensis*. Node labels refer to ML BS and Bayesian PP values. The time scale below the tree indicates million years ago.

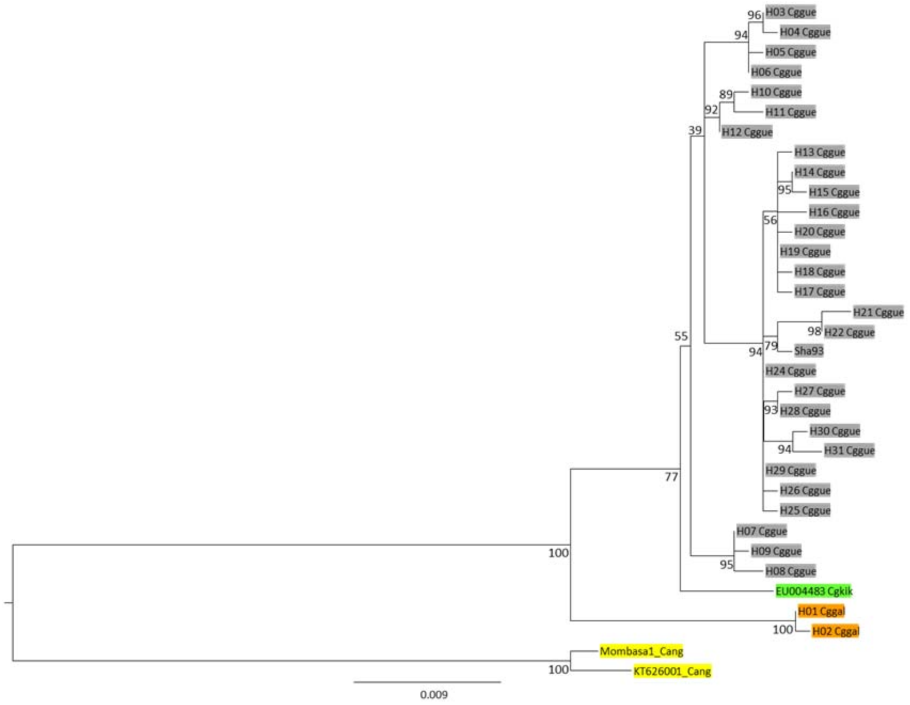

Figure S2b. ML tree (cytb). Csat = *Colobus satanas*, Cvel = *C. vellerosus*, Cpol = *C. polykomos*, Cang = *C. angolensis*, Cgocc = *C. guereza occidentalis*, Cggal = *C. g. gallarum*, Cgmat = *C. g. matschiei*, Cggue = *C. g. guereza*, Cgkik = *C. g. kikuyuensis*, Cgcau = *C. g. caudatus*, Cgue = *C. guereza* ssp.. Node labels refer to ML BS values.

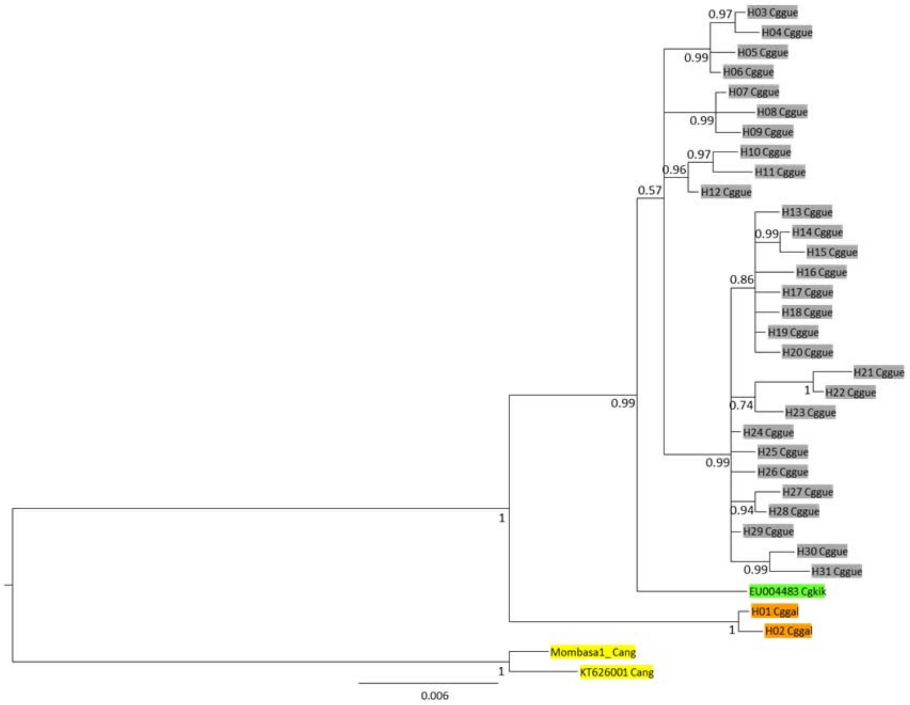

Figure S2c. Bayesian tree (cytb). Csat = *Colobus satanas*, Cvel = *C. vellerosus*, Cpol = *C. polykomos*, Cang = *C. angolensis*, Cgocc = *C. guereza occidentalis*, Cggal = *C. g. gallarum*, Cgmat = *C. g. matschiei*, Cggue = *C. g. guereza*, Cgkik = *C. g. kikuyuensis*, Cgcau = *C. g. caudatus*, Cgue = *C. guereza* ssp.. Node labels refer to Bayesian PPs.

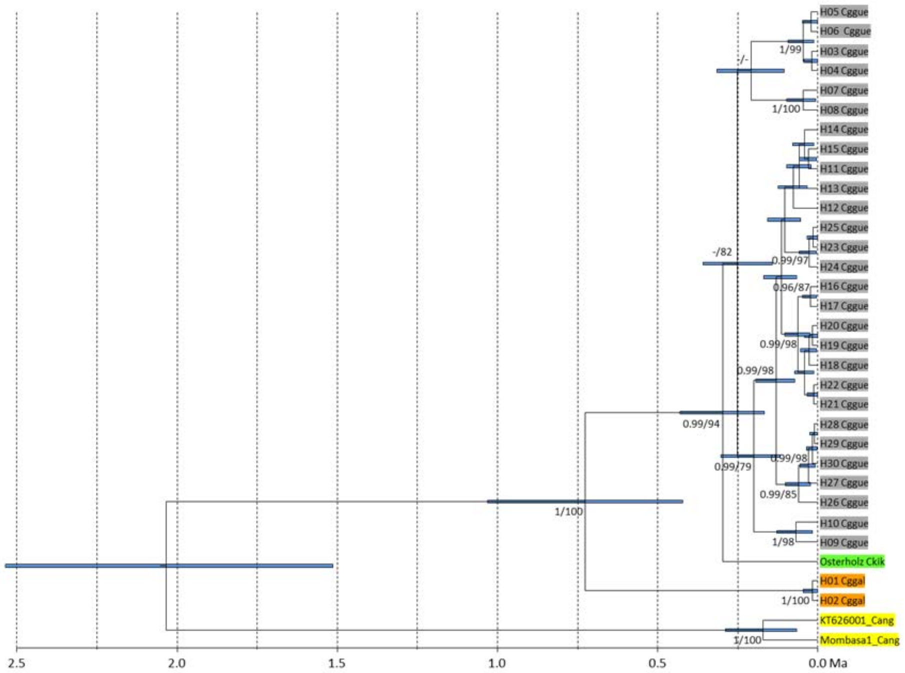

Figure S3a. Ultrametric tree showing phylogenetic relationships and divergence times of *Colobus* mtDNA lineages (based on concatenated 1930 bp of NADH and cytb). Tip labels refer to *Colobus* haplotypes (see Table S1). Cang = *C. angolensis*, Cggal = *C. g. gallarum*, Cggue = *C. g. guereza*, Cgkik = *C. g. kikuyuensis*. Node labels refer to ML BS and Bayesian PP values. The time scale below the tree indicates million years ago.

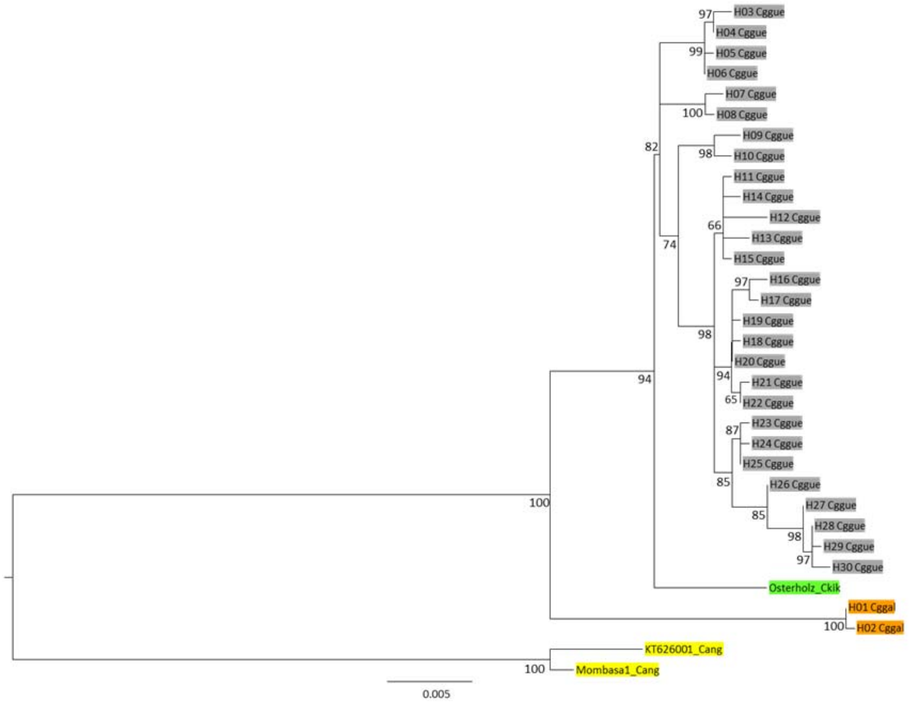

Figure S3b. ML tree (concatenated). Csat = *Colobus satanas*, Cvel = *C. vellerosus*, Cpol = *C. polykomos*, Cang = *C. angolensis*, Cgocc = *C. guereza occidentalis*, Cggal = *C. g. gallarum*, Cgmat = *C. g. matschiei*, Cggue = *C. g. guereza*, Cgkik = *C. g. kikuyuensis*, Cgcau = *C. g. caudatus*, Cgue = *C. guereza* ssp.. Node labels refer to ML BS values.

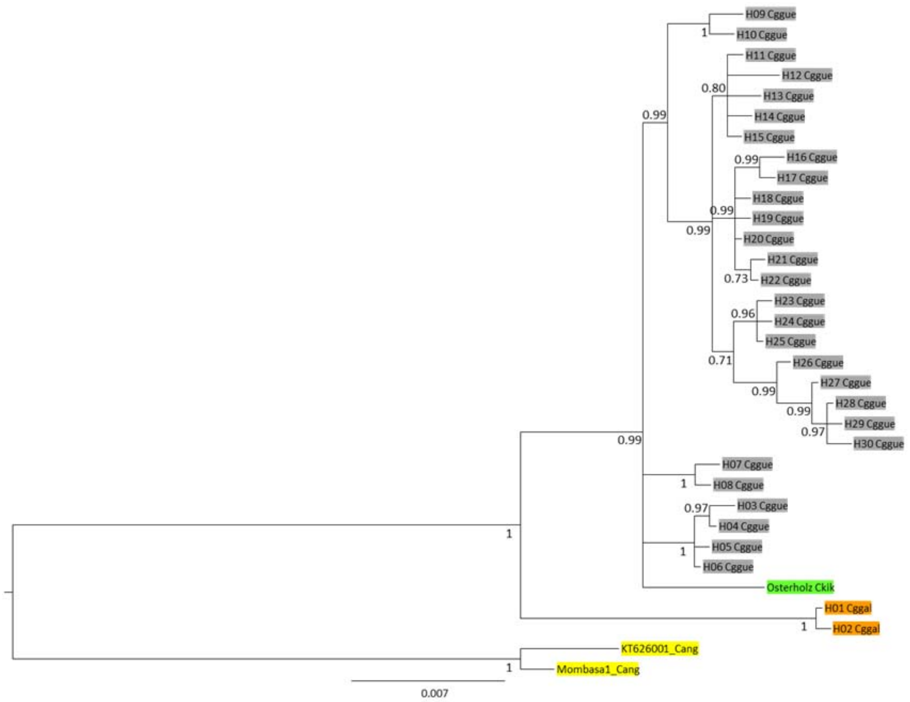

Figure S3c. Bayesian tree (concatenated). Csat = *Colobus satanas*, Cvel = *C. vellerosus*, Cpol = *C. polykomos*, Cang = *C. angolensis*, Cgocc = *C. guereza occidentalis*, Cggal = *C. g. gallarum*, Cgmat = *C. g. matschiei*, Cggue = *C. g. guereza*, Cgkik = *C. g. kikuyuensis*, Cgcau = *C. g. caudatus*, Cgue = *C. guereza* ssp.. Node labels refer to Bayesian PPs.

Table S1. Taxonomy, geographical origin (coordinates in decimal degrees) and haplotype assignments of *Colobus* samples and respective NCBI Accession numbers per haplotype.

taxon	ID	Country	Site	Lat	Long	NADH hap	Acc Num	cytb hap	Acc Num	comb hap
C. satanas		GNQ	Bioko Island			og	EU580054			
C. satanas		GNQ	Bioko Island			og	EU580055			
C. vellerosus		GHA	Boabeng-Fiema			og	EU580056			
C. polykomos		CIV	Tai National Park			og	EU580053			
C. angolensis	Mombasa1	KEN	s of Mombasa	-4.3353	39.5689	og	MK415322	og1	MK415348	og1
C. angolensis		KEN						og1	HQ859372	
C. angolensis			zoo, *palliatus*?			og	EU580047			
C. angolensis			unknown, zoo			og	EU580046			
C. angolensis			*palliatus*?			og	KT626001	og2	KT626001	og2
C. angolensis		KEN						og2	HQ859342	
C. g. matschiei?							AY863427			
C. g. matschiei		KEN	Kakamega Forest				EU580051			
C. g. matschiei			zoo?				DQ399681			
C. g. matschiei			zoo?				DQ399682			
C. g. matschiei			zoo?				DQ399685			
C. g. matschiei?			zoo?				DQ399683			
C. g. matschiei?			zoo?				DQ399684			
C. g. occidentalis			zoo?				DQ399697			
C. g. occidentalis			zoo?				DQ399698			
C. g. occidentalis		CMR	unknown locality				EU580052			
C. g. caudatus?			zoo, *caudatus*?				EU580049			
C. g. caudatus?			zoo?				DQ399690			
C. g. caudatus?			zoo?				DQ399692			
C. g. caudatus?			zoo?				DQ399695			
C. g. caudatus			zoo?				DQ399687			
C. g. caudatus			zoo?				DQ399688			
C. g. caudatus			zoo?				DQ399689			
C. g. kikuyuensis?			zoo, *kikuyuensis*?				EU580050			
C. g. kikuyuensis			zoo?				DQ399691			
C. g. kikuyuensis			zoo?				DQ399694			
C. g. kikuyuensis	Osterholz		zoo, *kikuyuensis*?				MK415323		EU004483	og
C. g. gallarum	ID1	ETH	Adas Mts Forest	9.3087	41.2449	h01	MK415324	h01	MK415349	h01
C. g.gallarum	ID2	ETH	Adas Mts Forest	9.3098	41.2453	h01		h01		h01

C. g. gallarum	ID3	ETH	Adas Mts Forest	9.3106	41.2453	h01		h02	MK415350	h02
C. g. gallarum	ID4	ETH	Adas Mts Forest	9.3106	41.2453	h01		h01		h01
C. g. gallarum	C1	ETH	Kuni-Muktart	9.0208	40.8402	h01		h01		h01
C. g. gallarum	C2	ETH	Kuni-Muktart	9.0258	40.8567	h01		h01		h01
C. g. gallarum	C3	ETH	Kuni-Muktart	9.0291	40.8424	h01				
C. g. gallarum	C4	ETH	Kuni-Muktart	9.0292	40.8550	h01		h01		h01
C. g. gallarum	C5	ETH	Kuni-Muktart	9.0106	40.8476	h01		h01		h01
C. g. gallarum	C6	ETH	Kuni-Muktart	9.0172	40.8303	h01		h01		h01
C. g. guereza	Ars07	ETH	Arussi	7.9853	39.6784	h02	MK415325	h03	MK415351	h03
C. g. guereza	Ars12	ETH	Arussi	7.9853	39.6784	h02		h03		h03
C. g. guereza	SH17	ETH	Kama1	7.3172	36.0702	h03	MK415326			
C. g. guereza	Ars04	ETH	Arussi	7.9853	39.6784	h04	MK415327	h05	MK415353	h05
C. g. guereza	Bns01	ETH	Benessa	6.3121	38.5000	h04		h03		h04
C. g. guereza	Bns08	ETH	Benessa	6.3121	38.5000	h04				
C. g. guereza	SH27	ETH	Boginda1	7.5256	36.1180	h04				
C. g. guereza	SH24	ETH	Kama1	7.3171	36.0704	h04				
C. g. guereza	Wondo26	ETH	Wondo Genet	7.0209	38.6362	h04		h06	MK415354	h06
C. g. guereza	Wondo31	ETH	Wondo Genet	7.0209	38.6362	h04		h06		h06
C. g. guereza	SH31	ETH	Yayoo	8.2895	35.6091	h04				
C. g. guereza	SH02	ETH	Boginda2	7.5094	36.1302	h05	MK415328			
C. g. guereza	SH13	ETH	Boginda2	7.5114	36.1217	h05				
C. g. guereza	SH07	ETH	Kayakela1	7.3202	36.2224	h06	MK415329	h11	MK415359	h07
C. g. guereza	Sha94	ETH	Shako, Bemch Maji	6.5000	35.0000	h06		h10	MK415358	h08
C. g. guereza	SH_ManII	ETH	Managesha II	8.9668	38.5455	h07	MK415330			
C. g. guereza	SH_ManIII	ETH	Managesha III	8.9668	38.5455	h07				
C. g. guereza	Bure25	ETH	Bure/Agew Mider	11.0180	36.7628	h08	MK415331	h18	MK415366	h13
C. g. guereza	Har06	ETH	Harenna	6.6827	39.8366	h09	MK415332	h22	MK415370	h29
C. g. guereza	Har07	ETH	Harenna	6.6827	39.8366	h09		h22		h29
C. g. guereza	Yir09	ETH	Yirgalem	9.7213	37.5724	h10	MK415333	h21	MK415369	h30
C. g. guereza	Bns02	ETH	Benessa	6.3121	38.5000	h10		h22		h28
C. g. guereza	Dale02	ETH	Dale Woreda, Kaffa	6.4499	38.2220	h10		h22		h28
C. g. guereza	SH36	ETH	Harenna 1	6.7476	39.7139	h10				
C. g. guereza	Yir12	ETH	Yirgalem	9.7213	37.5724	h10		h21		h30
C. g. guereza	Jma06	ETH	Jimma	7.6679	36.8861	h11	MK415334	h07	MK415355	h09
C. g. guereza	Jma67	ETH	Jimma	7.6679	36.8861	h11		h07		h09
C. g. guereza	Jma91	ETH	Jimma	7.6679	36.8861	h11		h07		h09
C. g. guereza	SH11	ETH	Boginda2	7.5117	36.1211	h12	MK415335	h22		h27

C. g. guereza	SH12	ETH	Boginda2	7.5112	36.1213	h12		h22		h27
C. g. guereza	SH18	ETH	Kama2	7.3193	36.0870	h13	MK415336			
C. g. guereza	SH21	ETH	Kama1	7.3110	36.0752	h14	MK415337			
C. g. guereza	SH08	ETH	Kayakela2	7.3233	36.2153	h15	MK415338			
C. g. guereza	SH03	ETH	Boginda2	7.5128	36.1206	h16	MK415339	h26	MK415374	h18
C. g. guereza	Bure28	ETH	Bure/Agew Mider	11.0180	36.7628	h16		h31	MK415379	h16
C. g. guereza	Bure32	ETH	Bure/Agew Mider	11.0180	36.7628	h16		h31		h16
C. g. guereza	Bure35	ETH	Bure/Agew Mider	11.0180	36.7628	h16		h31		h16
C. g. guereza	Bure37	ETH	Bure/Agew Mider	11.0180	36.7628	h16		h31		h16
C. g. guereza	Din1a	ETH	Dinibira, Kaffa	6.1500	37.3400	h16		h24	MK415372	h20
C. g. guereza	Din1b	ETH	Dinibira, Kaffa	6.1500	37.3400	h16		h30	MK415378	h17
C. g. guereza	SH22	ETH	Kama1	7.3145	36.0727	h16		h25	MK415373	h19
C. g. guereza	SH25	ETH	Kayakela2	7.3217	36.2138	h16		h26		h18
C. g. guereza	SH32	ETH	Yayoo	8.2895	35.6091	h17	MK415340	h29	MK415377	h21
C. g. guereza	Bale01	ETH	Bale Mts	7.2200	39.7175	h18	MK415341	h24		h22
C. g. guereza	Bale02	ETH	Bale Mts	7.2200	39.7175	h18		h24		h22
C. g. guereza	SH04	ETH	Boginda1	7.5170	36.1196	h18		h24		h22
C. g. guereza	WG06	ETH	West Gojam	11.3716	37.2656	h19	MK415342	h13	MK415361	h15
C. g. guereza	Woch02	ETH	Wochalle, Wollo	11.0251	39.6839	h19		h17	MK415365	h11
C. g. guereza	Woch33	ETH	Wochalle, Wollo	11.0251	39.6839	h19		h17		h11
C. g. guereza	Woch50	ETH	Wochalle, Wollo	11.0251	39.6839	h19		h17		h11
C. g. guereza	SH_WolIIA	ETH	Woliso	8.5348	37.9810	h19				
C. g. guereza	SH_WolIIB	ETH	Woliso	8.5348	37.9810	h19				
C. g. guereza	SH33	ETH	Bale Mts	7.0443	39.5463	h20	MK415343	h16	MK415364	h12
C. g. guereza	SH34	ETH	Bale Mts	7.0443	39.5463	h20		h16		h12
C. g. guereza	Bed35	ETH	Bedelle	8.4592	36.3618	h21	MK415344	h14	MK415362	h14
C. g. guereza	Bed48	ETH	Bedelle	8.4592	36.3618	h21		h14		h14
C. g. guereza	Bure48	ETH	Bure/Agew Mider	11.0180	36.7628	h21				
C. g. guereza	Jma01	ETH	Jimma	7.6679	36.8861	h21		h09	MK415357	h10
C. g. guereza	SH05	ETH	Kama1	7.3178	36.0695	h22	MK415345	h28	MK415376	h25
C. g. guereza	SH10	ETH	Boginda1	7.5331	36.1210	h22		h27	MK415375	h23
C. g. guereza	SH15	ETH	Boginda1	7.5301	36.1208	h22		h27		h23
C. g. guereza	Illi01	ETH	Illibabour	8.4879	35.6992	h22		h27		h26
C. g. guereza	Bed40	ETH	Bedelle	8.4592	36.3618	h23	MK415346			
C. g. guereza	SHBab	ETH	Boginda1	7.5331	36.1210	h24	MK415347	h28		h24
C. g. guereza	Ars11	ETH	Arussi	7.9853	39.6784			h04	MK415352	
C. g. guereza	Ars09	ETH	Arussi	7.9853	39.6784			h06		

C. g. guereza	Wondo32	ETH	Wondo Genet	7.0209	38.6362			h06		
C. g. guereza	SH06	ETH	Kama2	7.3231	36.0891			h07		
C. g. guereza	Jma02	ETH	Jimma	7.6679	36.8861			h08	MK415356	
C. g. guereza	Gesh02	ETH	Gesh	6.3689	35.1245			h12	MK415360	
C. g. guereza	Bed01	ETH	Bedelle	8.4592	36.3618			h15	MK415363	
C. g. guereza	Woch92	ETH	Wochalle, Wollo	11.0251	39.6839			h17		
C. g. guereza	Har15	ETH	Harenna	6.6827	39.8366			h19	MK415367	
C. g. guereza	Bure47	ETH	Bure/Agew Mider	11.0180	36.7628			h20	MK415368	
C. g. guereza	Dale01	ETH	Dale Woreda, Kaffa	6.4499	38.2220			h22		
C. g. guereza	Har03	ETH	Harenna	6.6827	39.8366			h22		
C. g. guereza	Har04	ETH	Harenna	6.6827	39.8366			h22		
C. g. guereza	Har08	ETH	Harenna	6.6827	39.8366			h22		
C. g. guereza	Har13	ETH	Harenna	6.6827	39.8366			h22		
C. g. guereza	Har22	ETH	Harenna	6.6827	39.8366			h22		
C. g. guereza	Illi02	ETH	Illibabour	8.4879	35.6992			h27		
C. g. guereza	Jma03	ETH	Jimma	7.6679	36.8861			h22		
C. g. guereza	Sha92	ETH	Shako, Bemch Maji	6.5000	35.0000			h22		
C. g. guereza	Sha93	ETH	Shako, Bemch Maji	6.5000	35.0000			h23	MK415371	

CIV = Ivory Coast, CMR = Cameroon, ETH = Ethiopia, GHA = Ghana, GNQ = Equatorial Guinea, KEN = Kenya; h = haplotype, og = outgroup haplotype

Table S2. Primers used in this study

**Primer ID**	**Sequence 5'-3'**
Cytb1-F	ACC AAT GAT ATG AAA AAY CAT
Cytb1-R	TGG TTG TAT AGT AGG GGT
Cytb2-F	ACA CCT ACT CTT TCT ACA
Cytb2-R	GTT TTG GGT ATT GGY GGT
NADH-F	CTA ACC CCT ACG AAT GCG G
NADH-R	CGT GAG GGA CTT TCA TTG TG
